# New Inverse Emulsion-Polymerized Iron/Polyaniline Composites for Permanent, Highly Magnetic Iron Compounds via Calcination

**DOI:** 10.3390/polym13193240

**Published:** 2021-09-24

**Authors:** Tar-Hwa Hsieh, Lin-Chia Ho, Yen-Zen Wang, Ko-Shan Ho, Cheng-Hsien Tsai, Li-Fan Hung

**Affiliations:** 1 Department of Chemical and Materials Engineering, National Kaohsiung University of Science & Technology, 415 Chien-Kuo Rd., Kaohsiung 80782, Taiwan; thh@nkust.edu.tw (T.-H.H.); chtsai@nkust.edu.tw (C.-H.T.); fish09331028@gmail.com (L.-F.H.); 2 Tri-Service General Hospital, 325 Sec. 2 Chenggong Rd., Neihu District, Taipei City 11490, Taiwan; 404010073@mail.ndmctsgh.edu.tw; 3 Department of Chemical and Materials Engineering, National Yu-Lin University of Science & Technology, 123, Sec. 3, University Rd., Dou-Liu City, Yun-Lin 64301, Taiwan

**Keywords:** inverse emulsion polymerization, polyaniline, superparamagnetic

## Abstract

The hydrophilic initiator potassium persulfate (KPS) was converted into a hydrophobic molecule by complexing with cetyltrimethylammonium bromide (CTAB) at both ends of the molecule (CTAPSu). Inverse emulsion polymerization thus proceeded inside micelles dispersed in the affluent toluene with CTAPSu as the initiator. Polyaniline (PANI) formed inside the micelles and entangled with Fe_3_O_4_ nanoparticles already esterified with oleic acid (OA). Iron composites consisted of OA-esterified Fe_3_O_4_ nanoparticles covered with PANI after de-emulsification. After calcination at 950 °C in an argon atmosphere, the resultant iron compound was a mixture of α-Fe (ferrite) and Fe_3_C (cementite), as determined by X-ray diffraction. Eventually, the calcined iron compounds (mixtures) demonstrated superparamagnetic properties with a high saturation magnetization (Ms) of 197 emu/g, which decayed to 160 emu/g after exposure to the atmosphere for four months.

## 1. Introduction

High-magnetic-moment materials are basic components of devices used in electronic, optical, and environmental fields. Moreover, they are used in the cell fabrication field as catalysts or electrodes since they can store power or hydrogen. More applications in medical areas include drug release and targeting, biosensors, imaging construction, and diagnosis of specific diseases.

Some of these applications need devices or instruments made of materials owning superparamagnetic properties and a high magnetic moment. The most common paramagnetic material is magnetite, usually obtained with the sol–gel method [1] at low temperature without calcination. However, its saturation magnetization (Ms) is below 100 emu g^−1^, i.e., whose magnetic moment is not high enough to meet the requirements of the applications. We have been working on preparing materials with Ms above 100 emu g^−1^ and focused on materials such as cementite (Fe_3_C) [2], ferric nitride (α″-Fe_x_N_y_) [3,4,5], and ferrite (α-Fe) [6], all of which have an Ms above 150 emu g^−1^.

Among high-magnetic-moment materials, ferrite is one of the best iron-related materials. However, ferrite is very unstable and can be oxidized to ferric oxide in few days in air. Using a convenient precursor (nano-Fe_3_O_4_), we are able to fabricate other iron-containing composites owning magnetic moment and capability of anti-oxidation via calcination at high temperature (>912 °C) in an inert gas, which induced rearrangement of Fe, C, and N atoms.

PANI(EB) (emeraldine form of polyaniline) is one of suitable long-chain polymers used to cover magnetic particles and able to provide both carbon and nitride sources for calcined compounds. There are also some other interesting applications concerning about calcined polyaniline-coated magnetic composites, such as EMI, microelectronics, medical treatments [7,8,9,10], etc. The eventual goal of different studies is to prepare a composite with a dominant ferrite core covered with hard/stable cementite or ferric nitride as the protecting material preventing further oxidation in the atmosphere.

PANI(EB) is usually prepared with the common emulsion polymerization [11,12,13,14,15] method in the affluent water, where aniline monomers (anilinium) are soluble. However, nano-Fe_3_O_4_ obtained from the sol–gel approach carries hydroxyl groups on the surface and can disperse well in water as well. The hydroxyl groups of nano-Fe_3_O_4_ can firmly associate with anilinium monomers in water before polymerization. Eventually, the resultant PANI (polyaniline) molecules are fully covered with Fe_3_O_4_ nanoparticles. Furthermore, Fe_3_O_4_ nanoparticles would remain on the surface of the nanofibrous PANI molecules [16,17,18,19,20,21,22,23]. The high-magnetic-moment nanoparticles obtained after high-temperature calcination remain on PANI surface rather than covered by PANI can easily oxidize in the atmosphere, which results in the loss of the magnetic moment. To introduce precursor Fe_3_O_4_ nanoparticles in PANI before calcination, an inverse emulsion system was designed, so to constraint both nano-Fe_3_O_4_ and anilinium monomers in surfactant-stabilized micelles of water in affluent toluene. To minimize the number of initiator molecules into the micelles in order to increase the molecular weight (MW) of the resultant PANIs, water soluble KPS (potassium persulfate) initiators are both end-capped with CTAB (cetylammonium bromide), converting it into more hydrophobic CTAPSu (cetylammonium persulfate). In fact, without this transformation, most of the hydrophilic initiators (KPS) would enter into the water micelles, and the system would be similar to that for inverse-suspension polymerization, leading to polymers with a lower MW and reducing the area of PANIs, which can are able to cover by the magnetic particles. Therefore, most of theCTAPSu are soluble in the toluene phase due to the long aliphatic wings on both ends of the molecule and only few molecules will diffuse into the water-containing micelles in which Fe_3_O_4_ and anilinium monomers are present. Consequently, the polymerization can proceed with only a limited amount of initiators in the micelles to ignite the polymerization, and high-MW PANIs can be obtained, which can firmly cover Fe_3_O_4_ nanoparticles inside and well-protect the formed iron composites from oxidation after calcination. A nano-Fe_3_O_4_/PANI composite via regular emulsion polymerization was also prepared for comparison.

The N- and C-doped iron composite before and after calcination at 950 °C in an argon atmosphere were characterized by FTIR (Fourier transform infrared spectroscopy) and X-ray diffraction (XRD) to analyze the composition of the obtained iron compounds (cementite, ferrite, or ferric nitride). We studied the morphologies of N- and C-doped iron-composites before and after calcination by SEM (scanning electron microscopy) and TEM (transmission electron microscopy).

## 2. Materials and Methods

### 2.1. Preparation

#### 2.1.1. Preparation of Nano-Fe_3_O_4_

We mixed 0.02 moles (4.33 g) of ferric chloride hexahydrate (FeCl_3_·6H_2_O, J.T. Baker, Phillipsburg, NJ, USA) and 0.01 mole (1.99 g) of ferrous chloride tetrahydrate (FeCl_2_·4H_2_O, J.T. Baker, Phillipsburg, NJ, USA) with 40 mL of deionized water in a beaker stirred with a magnetic stirrer. The resultant solution was transferred to a round-bottom three-necked flask that was equipped with a water condenser in one of the mouths. One of the remaining two mouths was purged with high-purity nitrogen to reduce the oxygen content above the atmosphere of the reaction mixture, the other one was the gas release outlet. The reaction solution was heated to 80 °C in a silicone oil bath for 10 min, with continuous supply of nitrogen. Then, some ammonia was introduced to adjust the alkalinity of the solution and start the reaction. The reaction continued for 30 min under magnetic stirring. Then, a powerful magnet was placed on the bottom of the reactor to attract the precipitate. The precipitate was washed several times with deionized water, and the clear solution in the upper layer was discarded. The isolated black precipitate was ultrasonicated in an oscillator for 20 min then dried in an oven for 12 h at 50 °C; eventually, nano-Fe_3_O_4_ was obtained [1,24].

#### 2.1.2. Preparation of Nano-Oleated Nano-Fe_3_O_4_ (Fe_3_O_4_(OA)) 

Fe_3_O_4_(OA) particles were prepared with the procedure previously described [1,24], except that 10 mL (0.03 mole) of OA was introduced before the addition of ammonium water, which was used to obtain an alkaline reaction mixture.

#### 2.1.3. Converting CTAB to CTAPSu

**Scheme 1:**
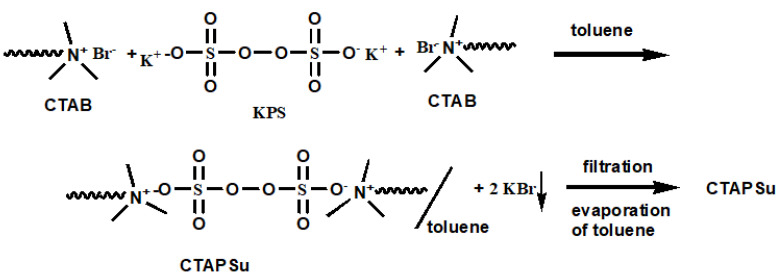
Preparation of CTAPSu.

The hydrophobic persulfate initiators for inverse emulsion polymerization were prepared by dissolving CTAB in water through stirring for 40 min before introducing a double amount of KPS to the water mixture, which was stirred for additional 40 min and then poured into a large amount of toluene. The precipitated KBr was removed by filtration, and the toluene solvent was evaporated in a vacuum hood. Eventually, a CTAPSu cake carrying two long alkyl wings was obtained (Scheme 1). The obtained CTAPSu cake was dried in a vacuum oven overnight.

#### 2.1.4. Preparation of PANI/Fe_3_O_4_ and PANI/Fe_3_O_4_(OA)) 

We dissolved 3 g (0.091 mol) n-dodecylbenzenesulfonic acid (HDBSA: Tokyo Kasei Kogyo Co., Tokyo, Japan) in 50 mL of de-ionized water. The mixture was slowly stirred until a homogeneous solution was obtained, followed by the addition of 9 g (0.0968 mol) of aniline (Tokyo Kasei Kogyo Co., Tokyo, Japan); the solution was stirred until clear. Eventually, Fe_3_O_4_ or Fe_3_O_4_(OA) obtained from the previous experiment [1,24] was added, and the mixture was stirred again until homogeneous so to obtain a water phase. The concentration of DBSA was about 1.82 M, well above the critical concentration (CMC) to create micelles.

We dissolved 2 g of CTAB and 19.4 g of CTAPSu prepared in Section 2.1.3 in 500 mL of toluene and poured only 5 mL of the water phase into the affluent toluene, with a volume ratio of 1:100. The inverse emulsified system was then created with anilinium monomers and Fe_3_O_4_ (or Fe_3_O_4_(OA)), which remained inside the micelles (inversely emulsified). After stirring, a W/O creamed solution formed, and the system was slightly heated to 40 °C and maintained for 3.5 h. The inverse emulsion polymerization of PANI was stopped by pouring 200 mL of acetone (de-emulsifier), and the composites (PANI(ES)/Fe_3_O_4_ or PANI(ES)/Fe_3_O_4_(OA)) were precipitated, isolated by filtration, and dried in an oven overnight.

The composites were then de-doped in the affluent 1M ammonium water by stirring for 2 to 3 days to remove the doping protonic acid and sulfide compounds derived from the dissociation of persulfate initiators. A de-doped composite cake (PANI(EB)/Fe_3_O_4_ or PANI(EB)/Fe_3_O_4_(OA)) was obtained from filtration and dried in an oven at 80 °C for one day. The wt% of Fe_3_O_4_ in the composite was about 40% according to the residue weight after 500 °C in the TGA thermogram in the presence of purging air.

A common emulsion polymerization experiment [25,26,27] was carried out with KPS as the initiator instead of CTAPSu with or without Fe_3_O_4_. Besides, HDBSA, which behaves as both surfactant and protonic acid in an inverse emulsion polymerization, was replaced by CTAB and HCl(aq). The obtained neat PANI and composite were called PANI(Em) (polyaniline prepared via regular emulsion polymerization) and PANI(Em)/Fe_3_O_4_, respectively. PANI prepared in such had a nanofibrous morphology [28,29].

#### 2.1.5. Calcination of the PANI/Iron Composites

PANI(EB)/Fe_3_O_4_ or PANI(EB)/Fe_3_O_4_(OA) prepared in Section 2.1.4 was calcined in a tube furnace ramping up to 950 °C at 10 °C min^-1^ and holding the temperature at 950 °C for 30 min in an argon atmosphere. The obtained N-, C-doped iron composites were called FeNC and FeNC(OA), depending on whether the precursor was Fe_3_O_4_ or Fe_3_O_4_(OA), respectively. FeNC was kept at room temperature for two months and then renamed FeNC-2. FeNC(OA) was named FeNC(OA)-2 or FeNC(OA)-4, respectively, after storage for two and four months at room temperature, respectively.

### 2.2. Characterization

#### 2.2.1. FTIR

The main functional groups of neat Fe_3_O_4_ and various iron-compounds were assigned according to the FTIR spectra that were recorded on an IFS3000 v/s FTIR spectrometer (Bruker, Ettlingen, Germany) at room temperature with a resolution of 4 cm^−^^1^ and 16 scanning steps.

#### 2.2.2. Ultraviolet–Visible–Near-IR Spectroscopy (UV–Vis–NIR)

The UV–Vis–NIR spectra of the samples were obtained with a Hitachi U-2001 and a DTS-1700 NIR Spectrometer (Nicosia, Cyprus). The wavelength used ranged from 300 to 1600 nm.

#### 2.2.3. TGA (Thermogravimetric Analysis)

The mass loss percentages of PANI(EB)/Fe_3_O_4_(OA) upon calcination (thermal degradation) was monitored and recorded using TGA (TA SDT-2960, New Castle, DE, USA).

#### 2.2.4. SEM

The size and morphology of various iron compounds were characterized by SEM (field-emission gun-scanning electron microscope, AURIGAFE, Zeiss, Oberkochen, Germany). The samples were prepared from strewn on carbonic tape and followed by posting on ferric stage. The samples were also surface-plated with gold in a CVD process to improve the surface conductivity and obtain a higher resolution.

#### 2.2.5. TEM

Photos of the samples were taken by a field-emission transmission electron microscope, HR-AEM (HITACHI FE-2000, Hitachi, Tokyo, Japan); the samples were dispersed in acetone before being placed on carbonic-coated copper grids dropwise before subjecting to the emission.

#### 2.2.6. EDS (Energy-Dispersive X-ray Spectra)

The EDS of various Pt catalysts were obtained using XL-40EFG, Philips (Eindhoven, The Netherland). The PANI(EB)/Fe_3_O_4_(OA) sample was coated with gold before measurement.

#### 2.2.7. Wide-Angled X-ray Diffraction (WXRD)

A copper target (Cu-Kα) Rigaku X-ray source (Rigaku, Tokyo, Japan) with a wavelength of 1.5402 Å was used for X-ray diffraction. The scanning angle (2θ) used was from 10° to 70° with a voltage of 40 kV and a current of 30 mA, operated at 1° min^−1^.

#### 2.2.8. X-ray Photoelectron Spectroscopy (XPS)

We applied the different binding energy spectra of N1s of neat PANI(EB) and various iron compounds to characterize various N-containing groups and Fe–N covalent bonding after calcination, using the XPS instrument Fison (VG)-Escalab 210 (Fison, Glasgow, UK) and an Al Ka X-ray source at 1486.6 eV. The pressure in the chamber was maintained under 10^−^^6^ Pa or lower during the measurement. A tablet-like sample was prepared by a stapler.

#### 2.2.9. Superconductor Quantum Interference Device (SQUID)

The magnetic properties of various iron compounds were measured by a Quantum Design MPMS-XL7 SQUID magnetometer (ISIS, San Diego, CA, USA)

## 3. Results and Discussion

This section is divided by subheadings. It provides a concise and precise description of the experimental results, their interpretation, as well as the experimental conclusions.

### 3.1. Subsection

#### FTIR Spectra

**Figure 1:**
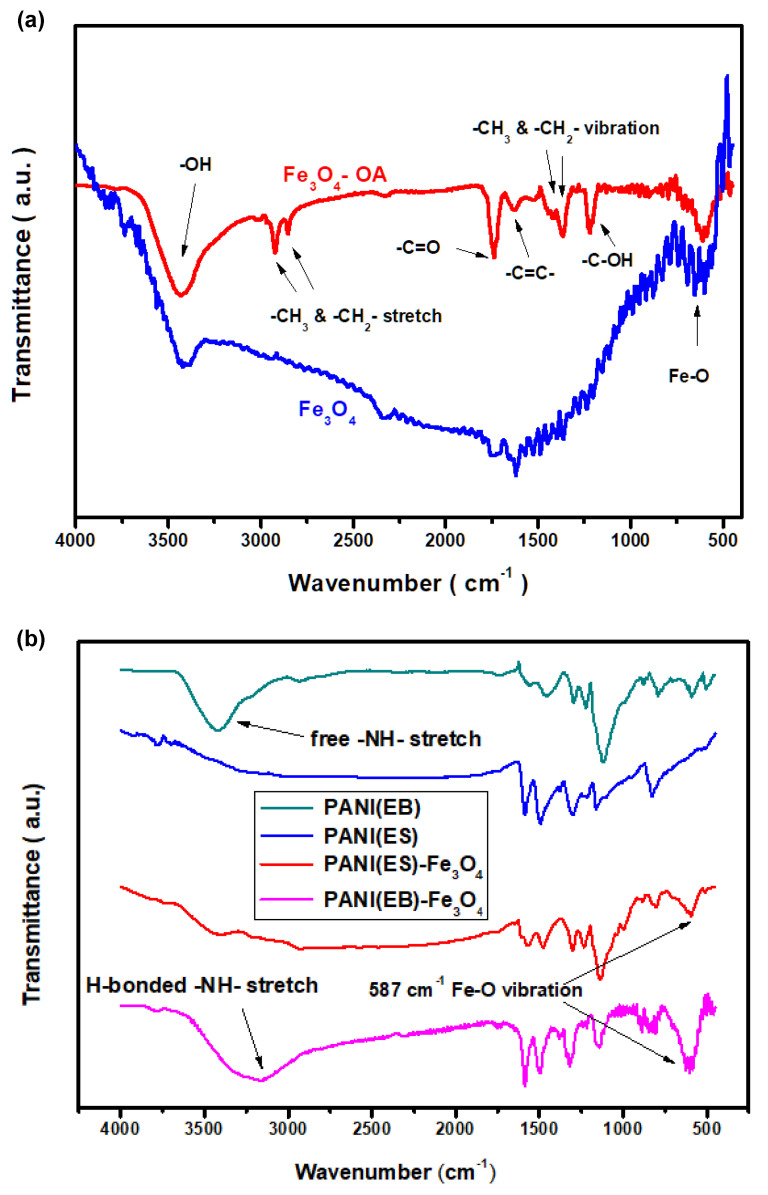
FTIR spectra of (**a**) Fe_3_O_4_ and Fe_3_O_4_(OA) (**b**) PANI and PANI/Fe_3_O_4_.

Fe_3_O_4_ nanoparticles prepared by the sol–gel method clearly showed hydroxyl groups at ~3300 cm^−1^ in accordance with Figure 1a, which did not disappear entirely after esterification with OA, as seen in Figure 1b. The presence of peaks around 587 cm^−1^ revealed the formation of Fe–O bonding; the symmetric and asymmetric stretching of aliphatic methylene and methyl groups of OA was clearly seen at 2920 and 2840 cm^−1^.

The prepared Fe_3_O_4_ nanoparticles were mixed with anilinium monomers before inverse emulsion polymerization. The obtained PANI(ES)/Fe_3_O_4_ composites were de-doped into PANI(EB)/Fe_3_O_4_ in NH_4_OH(aq), and their FTIR spectra were compared to those of neat PANI(ES) and PANI(EB), respectively, as shown in Figure 1b. The Fe–O groups were still present in the composites, as shown in Figure 1b, and the characteristic functional groups of PANI belonging either to ES or to EB, were also observed, even when the preparation was carried out in the presence of Fe_3_O_4_, indicating that the characteristic functional groups of PANI were not influenced by the presence of Fe_3_O_4._ However, some interaction occurred between PANI(EB) and Fe_3_O_4_ particles. For example, peaks belonging neither to neat PANI(EB) nor to Fe_3_O_4_ were observed at around 3160 cm^−1^, as shown in Figure 1b. Only single peak found for –NH– stretch mode (3417 cm^−1^) of PANI(EB) due to the H bonds with the –OH groups of Fe_3_O_4_, as shown in Figure 1a. The presence of H bonds between PANI(EB) molecules and Fe_3_O_4_ nanoparticles can modify the morphology of PANI(EB) in the composites, which will be examined by UV spectra.

### 3.2. UV–Vis Spectra

**Scheme 2:**
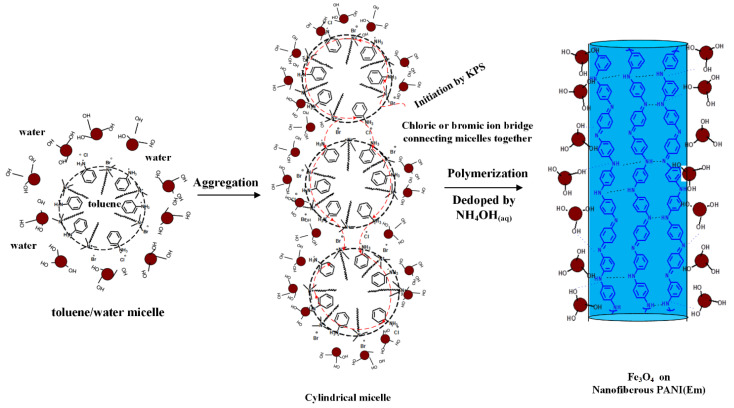
Preparation of the PANI(Em)/Fe_3_O_4_ composite via regular emulsion polymerization.

**Figure 2:**
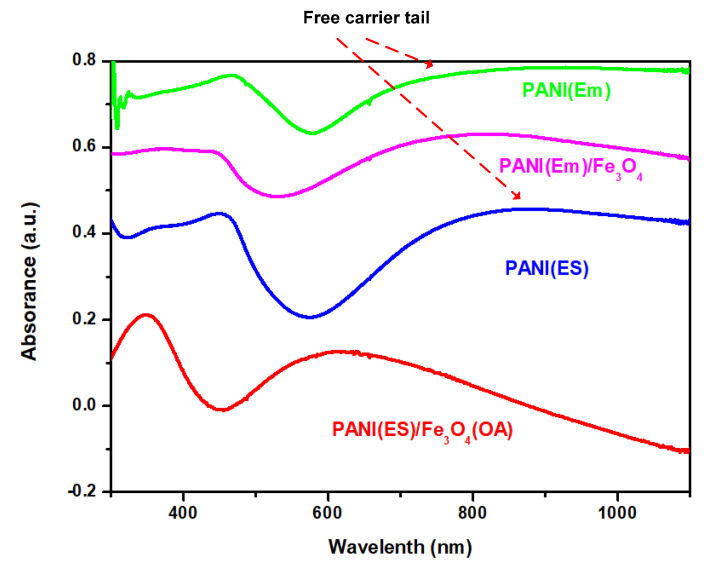
UV-Vis-NIR spectra of various PANIs.

The UV spectra of neat PANI and composites prepared with either common or inverse emulsion polymerization are shown in Figure 2. For neat PANI(Em) and PANI(ES), the so-called ‘free carrier tail’ [28,29], which is related to the increase of the conjugation chain length and super red-shift to the near-IR region due to the more extended PANI molecules, is demonstrated in Figure 2. After Fe_3_O_4_ nanoparticles were incorporated in PANI, the tails in the near-IR region decayed and became curved for samples prepared by both regular and inverse emulsion. The bended curves of the composites indicate the shortening of the conjugation (blue shift). The extended PANI molecules recoiled back appearing like random coils than rigid rods. The coiling driving force could originate from the H-bonding between the hydroxyl groups of Fe_3_O_4_ nanoparticles and the amino groups of PANI. For PANI(Em)/Fe_3_O_4_ prepared with the regular emulsion approach, the H-bonding mainly occurred on the rigid rod surfaces, and the nanoparticles remained on the surface, as described in Scheme 2, since the hydrophilic Fe_3_O_4_ nanoparticles would remain in the water phase rather than penetrate in the core of the micelles where polymerization occurred (Scheme 2).

**Scheme 3:**
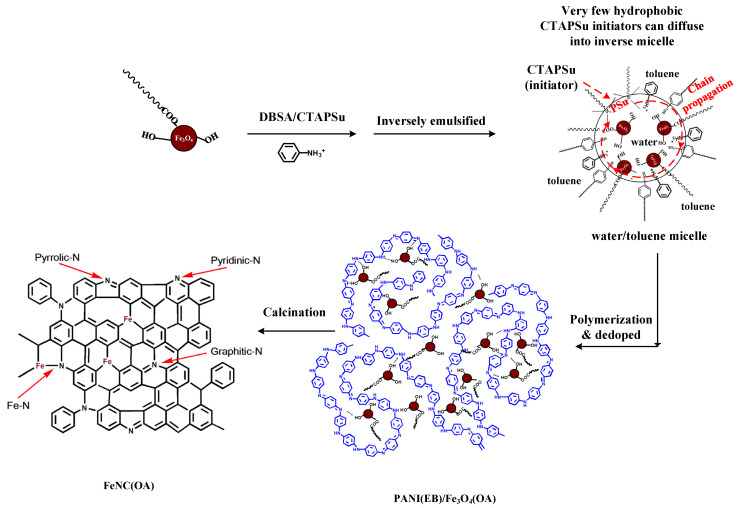
Preparation of the PANI/Fe_3_O_4_(OA) composite via inverse emulsion polymerization.

PANI(ES)/Fe_3_O_4_(OA) prepared with inverse emulsion also demonstrated a blue shift from the near-IR (890 nm) to the visible region (620 nm) for the λ_max_, revealing that hydroxylated Fe_3_O_4_ nanoparticles were able to enter the micelles where they were surrounded by the coiled PANI(ES) molecules after polymerization to de-dope polyaniline producing PANI(EB). The Fe_3_O_4_(OA) nanoparticles with surface–OH groups and only one oleic tail (based on the molar ratio described in Section 2.1.4) were capable of staying in the outer area of the micelles, where most of the anilinium monomers were located (Scheme 3) during the inverse emulsification process, covered by the coiled PANI(ES) after polymerization. Therefore, the significant blue shift of PANI(ES)/Fe_3_O_4_(OA) in the UV spectrum of Figure 2 came from the neutralization effect after contact with Fe_3_O_4_(OA), which turned PANI(ES)/Fe_3_O_4_(OA) to PANI(EB)/Fe_3_O_4_(OA), as depicted in Scheme 3. In contrast, the curve of the UV spectrum of PANI(Em)/Fe_3_O_4_ prepared via regular emulsion polymerization only demonstrated a slightly bending curve in the near-IR region after the introduction of Fe_3_O_4,_ because the obtained PANI rods were just covered by the Fe_3_O_4_ nanoparticles, as will be seen and discussed in the following SEM section.

### 3.3. TGA

**Figure 3:**
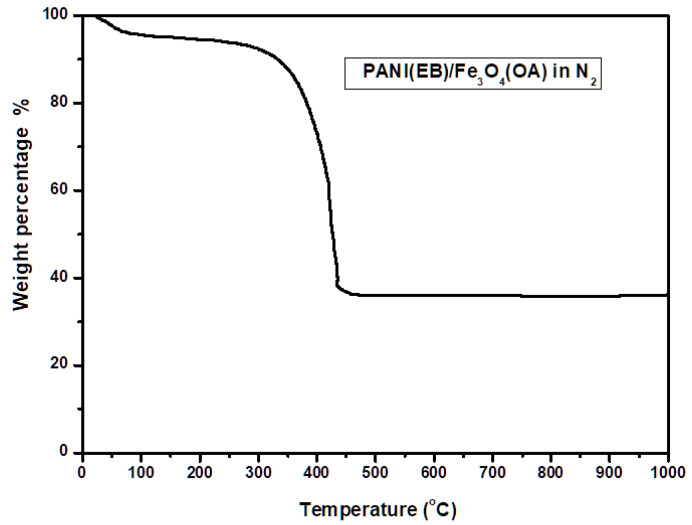
TGA thermogram of PANI(EB)/Fe_3_O_4_(OA).

The thermal degradation of PANI(EB)/Fe_3_O_4_(OA) was monitored by TGA. We observed about 5 wt% loss, as shown in Figure 3, due to the evaporation of water below 100 °C. A not significant weight loss was measured from 100 to 300 °C [15]. Crosslinking between neighboring PANI(EB) occurred at this stage, leading to the loss of only some H elements without causing significant weight loss as would result from breaking the backbones. After 300 °C, significant thermal degradation of PANI(EB) main chains and alkyl chains of OA occurred, and the matrix started to carbonize by driving some N element off in the form of ammonia and methane. The carbonized matrix became robust Fe, N-doped carbonaceous composites after 500 °C, and weight loss was negligible up to 1000 °C. Eventually, a residual weight of 35% was measured, which is well above the theoretical value of iron, indicating some of the PANI(EB) was retained in the form of a Fe, N-doped composite after calcination, as described in the final portion of Scheme 3.

### 3.4. SEM

**Figure 4:**
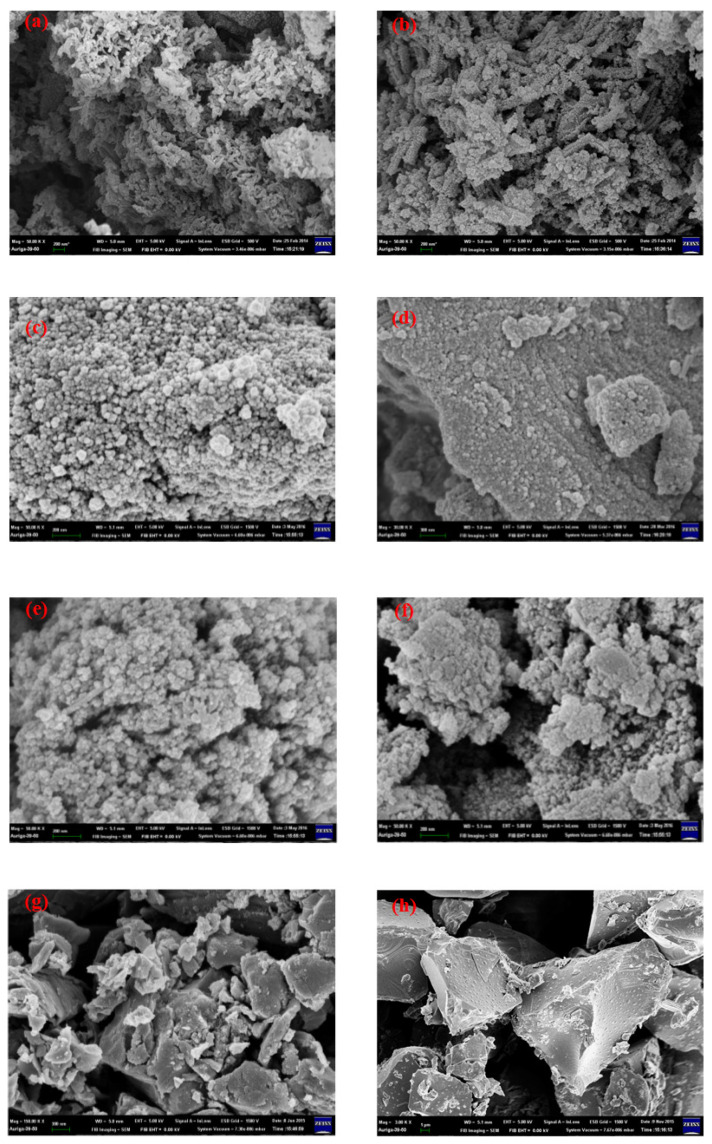
SEM micro-pictures of PANI and various iron compounds (**a**) PANI(Em) (**b**) PANI(Em)/Fe_3_O_4_ (**c**) Fe_3_O_4_ (**d**) Fe_3_O_4_(OA) (**e**) PANI(EB)/Fe_3_O_4_ (**f**) PANI(EB)/Fe_3_O_4_(OA) (**g**) FeNC (**h**) FeNC(OA).

For PANI(EB) prepared with the regular emulsion approach, a nanofibrous morphology was clearly observed in SEM micro-pictures (Figure 4a). The nanofibrous morphology originated from the interconnection of micelles by counter ions before polymerization [28], resulting in the rigid-rod morphology after polymerization, which is commonly found in the emulsion polymerization of PANI, as described in Scheme 1 [29].

Due to their hydrophilic nature, hydroxylated Fe_3_O_4_ particles (referring to the FTIR spectrum in Figure 1) would remain outside the micelles in the water phase before and after the polymerization, covering the rigid-rod surfaces of PANI eventually (Figure 4b). Comparing the sizes of the PANI nanofibers without and with Fe_3_O_4_ particles (Figure 4a,b) present during polymerization, the diameter of the PANI nanofibers increased from 100 to 200 nm when many Fe_3_O_4_ particles covered on fibers’ surface.

However, PANI(Em)/Fe_3_O_4_ obtained from common emulsion was only covered with Fe_3_O_4_ nanoparticles, which can be easily converted to ferrite after calcination, and the unprotected ferrite can be easily oxidized into either Fe_3_O_4_ or cementite when exposed to the open air; in addition, the lower magnetization can largely decrease the bulk magnetization of the calcined iron composites. In other words, the iron-enriched ferrite composite with a high magnetic moment needs the protection of a calcined PANI covering to prevent the oxidation or the diffusion of carbon into the ferrite matrix, resulting in the formation of cementite. The evidence of the conversion from ferrite to either cementite or oxidized Fe_3_O_4_ was provided by the X-ray diffraction patterns of the calcined products.

Both Fe_3_O_4_ and Fe_3_O_4_(OA) particles demonstrated a connected pearl-like morphology, as shown in Figure 4c,d. The strong interaction (mainly H-bonding) between these particles and anilinium monomers in the outer area of the micelles during inverse emulsification allowed the obtained PANI molecules to adopt a curved conformation with a reduced crystalline structure. The rigid-rod morphology was no more seen in either PANI(EB)/Fe_3_O_4_ or PANI(EB)/Fe_3_O_4_(OA) and was mostly replaced by particular morphologies, in accordance with Figure 4e,f and as described in Scheme 3. The calcined composites (FeNC) demonstrated a largely curved slab-like morphology due to the gradual carbonization of the polyaniline matrix with the proceeding of calcination, which fused the smaller particles into huge slabs, as seen in Figure 4g.

### 3.5. TEM Micropictures

**Figure 5:**
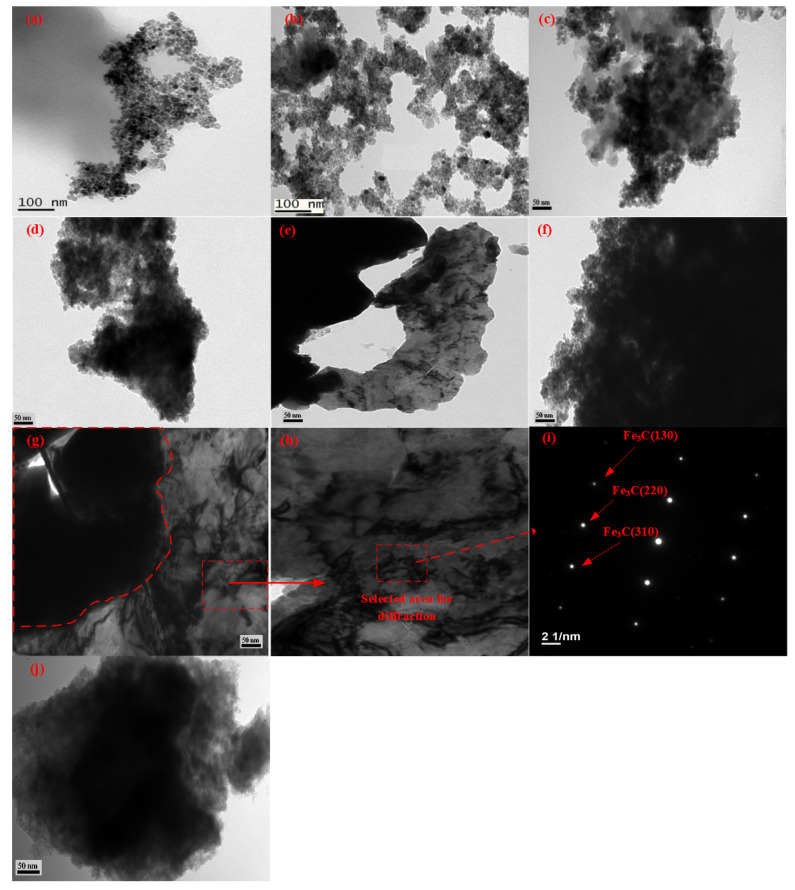
TEM micro-pictures of (**a**) Fe_3_O_4_ (**b**) Fe_3_O_4_(OA) (**c**) PANI(EB)/Fe_3_O_4_ (**d**) PANI(EB)/Fe_3_O_4_(OA) (**e**) FeNC (**f**) FeNC(OA) (**g**) FeNC-2 (**h**) FeNC-2 (enlarged) (**i**) e-diffraction pattern of vein-like texture (**j**) FeNC(OA)-2.

Similar to the SEM micro-pictures in Figure 4, the TEM micro-pictures of neat Fe_3_O_4_ and Fe_3_O_4_(OA) demonstrated the presence of pearl-like nanoparticles with similar sizes, as seen by comparing Figure 5a with Figure 5b. The presence of OA during the preparation of Fe_3_O_4_ seemed to fasten the aggregation of the nanoparticles, as shown in Figure 5b. The same phenomenon can be seen in Figure 5c,d for PANI/Fe_3_O_4_ and PANI/Fe_3_O_4_(OA) mixtures which were obtained via inverse emulsion polymerization. The graft of OA on Fe_3_O_4_ (Fe_3_O_4_(OA)) surfaces stabilized the micelle and allowed more Fe_3_O_4_ nanoparticles in the micelles. Besides, the swelling micelles resulting from the larger and more stable polymerizing PANI molecules (polymer droplets), prevented the early precipitation of PANI/Fe_3_O_4_(OA) from the broken polymer droplets, as described in Scheme 3.

After calcination at 950 °C, all pearl-like Fe_3_O_4_ particles disappeared and fused into clusters (Figure 5e,f) due to the carbonization of polyaniline matrix. Possibly, some of the α-Fe would converted to Fe_3_C, combining with the carbon provided by PANI during calcination, developing into a veined texture, present around or inside a dark cluster, as shown in Figure 5e for FeNC. However, for FeNC(OA) (Figure 5f), almost no veined texture in the dark cluster could be seen after calcination, indicating the formed α-Fe was well mixed with Fe_3_C, which has a very dense structure and can effectively hinder O_2_ from diffusing into the α-Fe domains and maintain its high magnetic moment.

The density of Fe_3_C is 7.69 gcm^−3^, which is almost equivalent to that of α-Fe (7.87 g cm^−3^), scattering TEM electrons equally. Therefore, most of the α-Fe are staying with some Fe_3_C in the dark area of TEM pictures of FeNCs, whose vein texture are not perceivable due to the similar electron scattering capability. The two types of iron compounds (α-Fe/and Fe_3_C) were the dominant species, and less Fe_3_O_4_ (density is 5.17 gcm^−3^) was present after calcination at 950 °C, based on the following discussions on the X-ray diffraction and SQUID spectra.

The morphology of FeNC changed gradually, and more veined textures of Fe_3_C appeared after two months in the atmosphere, as observed when comparing Figure 5e with Figure 5g or Figure 5h. The e-diffraction pattern demonstrated in Figure 5i clearly shows the vein-like textures contributed by Fe_3_C. Obviously, the unprotected α-Fe of FeNC could be converted into Fe_3_C gradually even at room temperature if it stayed in the atmosphere for enough time. Eventually, the magnetization of FeNC-2 would decrease by sacrificing α-Fe to Fe_3_C, which we will discuss in the following X-ray diffraction and SQUID. However, for FeNC(OA), the morphology was almost the same after two months, as seen when we compare Figure 5f with Figure 5j. The vein-like morphology around the dark cluster was not significant, as shown in Figure 5j, indicating that α-Fe was still the dominant species in the dark cluster, maintaining its high magnetic moment. The presence of some Fe_3_C in the dark cluster can protect the FeNC(OA) from oxidation during long-term exposure to the open air. It is still possible that some of the α-Fe will be oxidized to Fe_3_O_4_ after long-time exposure in the open air if the protecting Fe_3_C is phase-separated from the dark cluster, leaving the vulnerable α-Fe to O_2,_ like FeNC and FeNC-2, which will be confirmed and discuss later.

### 3.6. EDS

**Figure 6:**
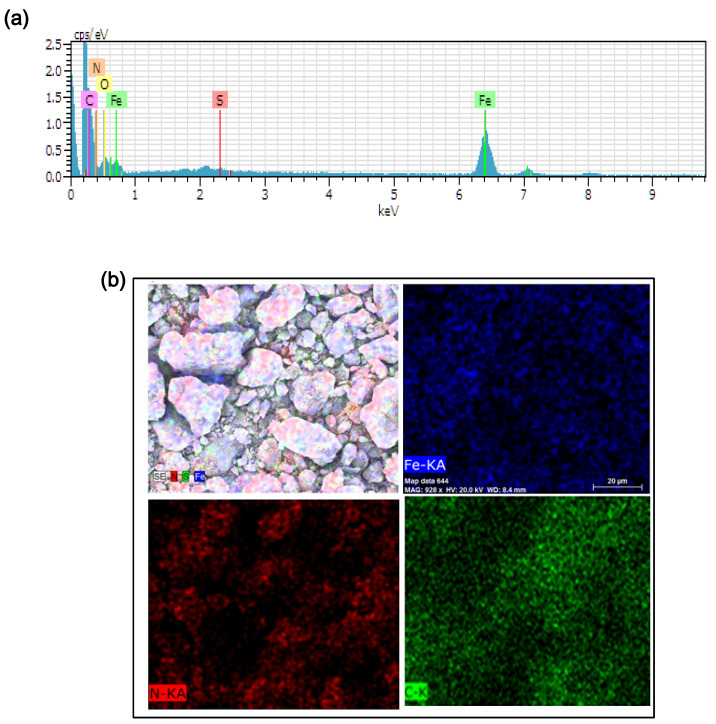
EDS (**a**) spectrum (**b**) Fe, N, and C mapping of FeNC(OA).

The EDS spectrum of FeNC(OA) and its element mappings are illustrated in Figure 6a,b, respectively. Figure 6a clearly demonstrates the presence of Fe, N, and C elements, C is the dominant element, and both Fe and N peaks are clearly seen. The sulfur element derived from KPS, which was converted to sulfate ion after initiating the polymerization of polyaniline. The element mappings demonstrated in Figure 6b revealed the presence of Fe, N, and C elements (in the form of α-Fe, Fe_4_N, or Fe_3_C), uniformly distributed in the carbonaceous matrix. The sulfur in Figure 6a came from the sulfate salt which formed from APS after initiating the polymerization of aniline salts.

### 3.7. XRD Patterns

**Figure 7:**
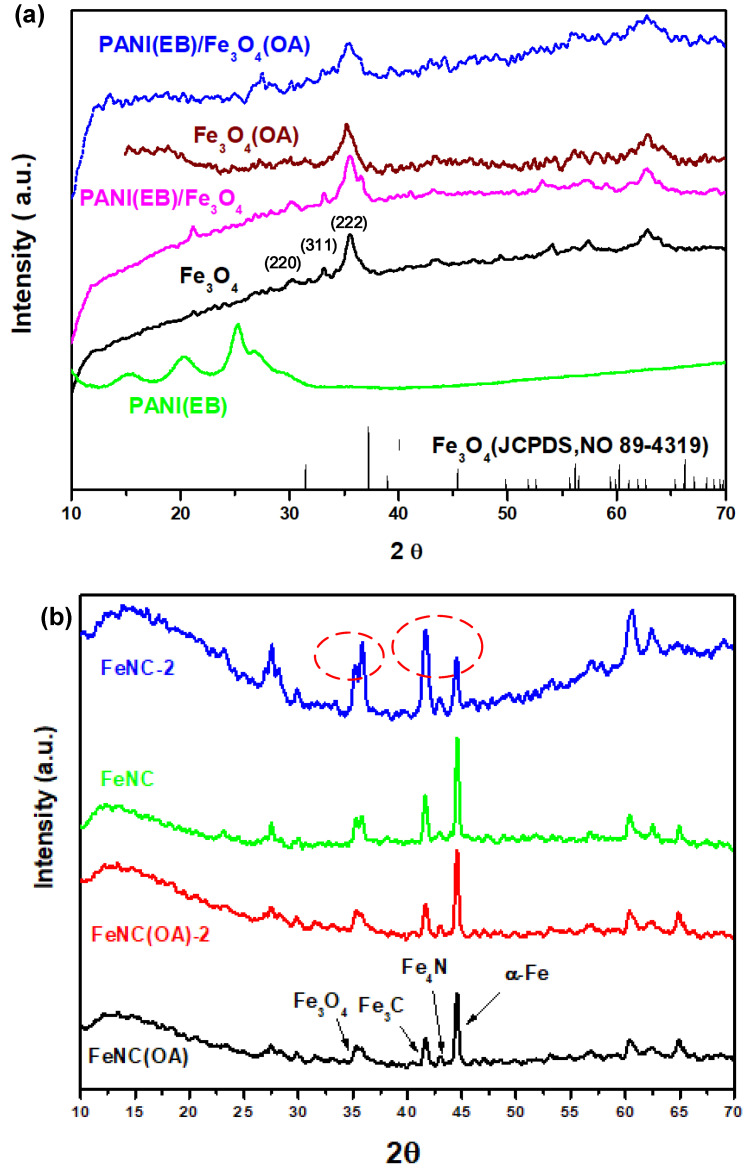
X-ray diffraction patterns of (**a**) PANI and iron compounds before calcination (**b**) iron compounds after calcination.

The X-ray diffraction patterns of neat PANI(EB) and nano-Fe_3_O_4_ compounds are displayed in Figure 7a, which demonstrates the crystalline patterns of polyaniline and Fe_3_O_4_ before and after mixing via polymerization. The pattern of neat Fe_3_O_4_ matches well with the standard pattern of Fe_3_O_4_ (JCPDS, No. 89-4319); smoother curves were found in the spectrum, indicating higher crystallinity. A clear pattern of peaks smaller than 30° (2θ) were seen for neat PANI(EB) due to the high degree of crystallization. However, peaks with some noise signals were present in the patterns of composite materials, especially after Fe_3_O_4_ was modified by OA before polymerization. Usually, Fe_3_O_4_ crystals are easily destroyed when a long-tail molecule like OA is attached on the surface, which might contribute to the imperfect diffracted pattern seen in the spectrum. Besides, no significant characteristic diffraction peak was found for PANI in PANI(EB)/Fe_3_O_4_(OA) composites, indicating that the crystalline structure of PANI(EB) in the PANI(EB)/Fe_3_O_4_(OA) was almost entirely demolished by the presence of Fe_3_O_4_(OA) nanoparticles in the micelles.

Significant peaks at 44° of α-Fe are visible in the X-ray spectra of Figure 6b after calcination at 950 °C for all iron-nanocomposites, contributing to the excellent high Ms (>120 emu g^−1^), which will be discussed later.

These peaks are characteristic diffraction peaks of cementite (Fe_3_C) and ferrite (α-Fe), respectively, with a higher Ms than 120 emu g^−1^, even reaching 197 emu g^−1^ for α-Fe. The magnetic moment contributed from Fe_3_C could be retained for a long time due to the perfect, hard crystalline structure, which could effectively prevent further oxidation in the atmosphere. However, α-Fe, which is made of pure Fe element, can easily be oxidized or converted to other iron compounds with weaker magnetization if it stays in the atmosphere for a long time. For example, soft α-Fe can combine with oxygen in the air and become Fe_3_O_4_ (oxidation) again, and the carbons close to α-Fe can also diffuse into the soft α-Fe matrix and create the harder crystalline structure of Fe_3_C. Both types of reactions turn out to be products with a lower magnetic moment compared to α-Fe and would significantly decrease the bulk magnetization. When we compared the X-ray diffraction patterns obtained two months later with the original one (Figure 7b), we noticed that iron composite prepared with neat Fe_3_O_4_ (FeNC) could develop into a compound with more Fe_3_O_4_ and Fe_3_C (42°). Based on the TEM micro-pictures (Figure 5g,h), we found many veined textures developed only by the sides of FeNC-2 after two months. 

The TEM picture (Figure 5j) shows that no significant Fe_3_C precipitated from the matrix compound during two month in the air, indicating some hard, perfect crystallized Fe_3_C mixed with α-Fe can effectively prevent oxidation from losing its magnetic moment.

### 3.8. XPS Spectra

**Figure 8:**
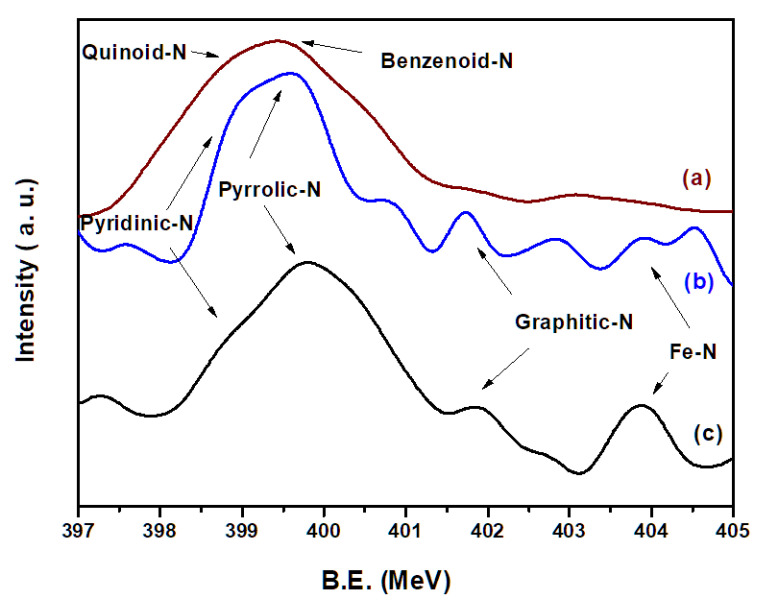
XPS spectra of N_1s_ of (**a**) PANI(EB) (**b**) FeNC (**c**) FeNC(OA).

The XPS of N_1s_ of neat PANI(EB), FeNC, and FeNC(OA) are illustrated in Figure 8 and characterize the possible N-containing products after calcination. In Figure 8a, no complicated N-related groups are seen, but quinoid-N and benzenoid-N are observed, which are connected alternatively to become PANI(EB). However, various N-containing groups [30,31,32] include pyridinic N (six-membered ring present in the edge), pyrrolic N (five-membered ring present on the edge), graphitic N (six-membered ring present in the middle of graphene-like matrix), and Fe–N emerged after calcination at 950 °C, and are depicted in the last portion of Scheme 3. Both FeNC (Figure 8b) and FeNC(OA) (Figure 8c) contain a large quantity of pyrrolic N and less graphitic N.

### 3.9. SQUID Spectra

Calcination above 900 °C for the blended mixtures of polyaniline and nano-Fe_3_O_4_ provided enough energy for the movement of various elements including Fe, N, C, and O. The arrangement of these elements created a new form of iron-related compounds. All of them demonstrated much higher Ms than the original Fe_3_O_4_ and contributed to the high Ms due to the presence of Fe_3_C, Fe_4_N, and α-Fe in the calcined composites.

**Figure 9:**
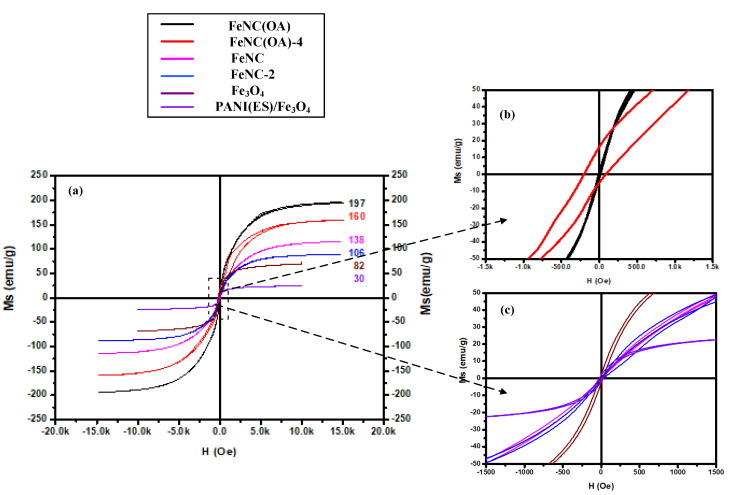
(**a**) SQUID curves for various iron compounds. (**b**,**c**) are enlarged insets of (**a**), respectively.

As expected, FeNC and FeNC(OA), which were obtained from the calcination of PANI(EB)/Fe_3_O_4_ and PANI(EB)/Fe_3_O_4_(OA) at 950 °C, respectively, demonstrated high saturated magnetization of 138 and 197 emu g^−1^ compared to 82 emu g^−1^ of neat Fe_3_O_4_ and 30 emu g^−1^ of PANI(ES)/Fe_3_O_4_, in accordance with Figure 9a. The high magnetic moment of FeNC and FeNC(OA) derived from the formation of both α-Fe and Fe_3_C, as already confirmed in the section of X-ray diffraction patterns. However, the Ms of FeNC decayed by 23.2%, from 138 to 106 emu g^−1^ in the air within two months, according to Figure 9a. For FeNC(OA), the decay was about 18.7%, from 197 to 160 emu g^−1^, still less than that of FeNC (23.2%) after a longer time (four months) at room temperature.

Regularly, the steep loss of magnetic moment of α-Fe derived from the oxidation in air when it could not convert to other protective iron compounds, and low-magnetic-moment iron oxides became the main products. For either FeNC or FeNC(OA), a not significant increase of Fe_3_O_4_ was observed after two and four months in air, respectively (i.e., FeNC-2 and FeNC(OA)-4) from their X-ray diffraction spectra. Therefore, the magnetic moment was lost mostly due to the transformation from high-magnetic-moment α-Fe to Fe_3_C, since not significant oxidation can occur in the air under the protection of the carbonized PANI. In Figure 7b of the X-ray pattern, an increasing peak of Fe_3_C and a decreasing peak of α-Fe were observed only for FeNC-2, whereas such phenomenon was not observed for FeNC(OA)-2 which still had a high magnetic moment two months later. For FeNC-2, more newly formed Fe_3_C precipitated from the matrix (Figure 5g). Hard materials like Fe_3_C mixed with FeNC(OA)-2 can effectively prevent further carbon diffusion into the inner α-Fe matrix and stop the advanced conversion to Fe_3_C. In other words, attaching a long alkyl chain to the surface of Fe_3_O_4_ nanoparticles via the inverse emulsion polymerization of PANI enhanced and stabilized the magnetization of the resultant C, N-doped iron compounds (FeNC(OA)). They successfully developed into dominant α-Fe via calcination under the protection of the surrounding PANI molecules and maintained a high magnetic moment in air under the protection of some hard and stable Fe_3_C.

All samples demonstrated perfect superparamagnetivity with slight hysteresis and not significant residual magnetivity, as shown in Figure 9a. Though some of the magnetization could be maintained after four months for FeNC(OA)-4, hysteresis was enhanced according to Figure 9b (upper inset diagram of Figure 9a); hysteresis was smaller for FeNC-2, as shown in Figure 9c (down inset diagram of Figure 9a).

## 4. Conclusions

OA tails were successfully attached to Fe_3_O_4_ (Fe_3_O_4_(OA)) nanoparticles by esterification, as revealed by IR spectra, which allowed them to be collected inside micelles during inverse emulsion polymerization. The strong blue shift of the UV–Vis–NIR spectrum of PANI(ES) demonstrated Fe_3_O_4_(OA) was well mixed with PANI molecules formed inside micelles during polymerization. The larger size of the particles seen in the SEM micro-pictures indicated the strong magnetic moment of FeNC(OA). TEM micrographs indicated the presence of Fe_3_C after calcination, and the new-borne Fe_3_C either phase-separated or remained in the matrix of α-Fe after exposure to air for two months. X-ray diffraction patterns of the iron compounds after calcination revealed the formation of Fe_3_C by sacrificing α-Fe to Fe_3_C gradually in air, if it was not protected by the new-borne Fe_3_C. The high, stable saturated magnetization (197 emu g^−1^) of FeNC(OA) resulted from affluent α-Fe and demonstrated negligible decay in two months as a result of the protection of by new-borne Fe_3_C.
